# Double Trouble! Do Workplace Supports Mitigate Lost Productivity for Young Workers with Both Severe Rheumatic Diseases and Depressive Symptoms?

**DOI:** 10.1007/s10926-024-10217-8

**Published:** 2024-07-03

**Authors:** Kathleen G. Dobson, Monique A. M. Gignac, Lori Tucker, Arif Jetha

**Affiliations:** 1 Institute for Work and Health, Suite 1800 400 University Avenue, Toronto, ON M5G1S5 Canada; 2 Dalla Lana School of Public Health, University of Toronto, Toronto, ON Canada; 3 University of British Columbia, Vancouver, BC Canada; 4 BC Children’s Hospital, Vancouver, BC Canada

**Keywords:** Rheumatic arthritis, Depression, Comorbidity, Productivity, Absenteeism, Workplace support

## Abstract

**Background:**

The objectives of this longitudinal study were to understand how comorbid rheumatic disease and depression symptoms were associated with at-work productivity among young adults, and to examine whether workplace support modified this association.

**Methods:**

Seventy-six Canadian young adults who were employed and living with a rheumatic disease were surveyed three times over 27 months. Morbidity was defined by whether participants reported severe rheumatic disease symptoms and/or depressive symptoms. Participants were asked about presenteeism, absenteeism, and whether the workplace support needs (accommodation and benefit availability and use) were met. Generalized estimating equations were used to address study objectives.

**Results:**

Seventeen participants experienced neither severe rheumatic disease nor depressive symptoms (no morbidity), 42 participants experienced either severe rheumatic disease or depressive symptoms (single morbidity), and 17 participants reported comorbidity at baseline. Participants with comorbidity reported greater presenteeism scores and were most likely to report absenteeism, compared to the other two morbidity levels. Having workplace support needs met was associated with decreased presenteeism over the 27-month period among participants with no and a single morbidity. Conversely, unmet support need was associated with greater presenteeism for participants with comorbidity. Having workplace support needs met did not modify the association between morbidity and absenteeism.

**Conclusion:**

Comorbid rheumatic disease and depression burden reduce productivity among young adults. A supportive work environment has the potential to address at-work productivity challenges. Additional research is needed to understand how workplace supports coupled with clinical interventions may tackle challenges at work for young adults living with rheumatic disease and depression.

**Supplementary Information:**

The online version contains supplementary material available at 10.1007/s10926-024-10217-8.

## Introduction

Supporting the work productivity of young adults are important economic, clinical, and public health policy priorities. Higher work productivity can benefit a young worker by offering opportunities for career growth and employment stability, and strengthen pathways to resources and supports that can help achieve better health in later adulthood [1]. Having a rheumatic disease can disrupt work productivity in young adulthood and increase the need for workplace supports [2] to be able to maintain employment. Experiencing depression symptoms is also common among young adults with rheumatic disease and may exacerbate the productivity difficulties faced by young workers with rheumatic disease [3–6]. In this article, we present findings from a longitudinal study of young adults living with rheumatic disease with and without depressive symptoms to examine productivity at work and the role of workplace supports in changes to absenteeism and presenteeism.

Productivity loss—including absenteeism (i.e., health-related missed workdays) and presenteeism (i.e., working while unwell)—is a marker of work disability and may adversely impact sustained employment participation and career advancement [6–8]. For young adults, lost productivity can lead to economic scars that may adversely affect employment experiences and opportunities later in life [9]. Rheumatic disease in young adulthood is associated with symptoms such as pain and fatigue that can interrupt productivity at work [10–12]. For instance, in a survey of 143 Canadian young adults living with different rheumatic diseases—which included juvenile arthritis, rheumatoid arthritis, and systemic lupus erythematosus—over 40% of participants reported lost productivity [2]. Findings from this survey and several other studies indicated that decreased productivity was associated with more severe rheumatic disease symptoms [13–15].

A supportive work environment which provides job accommodations and benefits can improve productivity among young adults living with rheumatic disease [16–20]. Job accommodations can include opportunities to modify job tasks and scheduling flexibility, while benefits include access to extended healthcare benefits or sick days. Recent cross-sectional research among young adults living with rheumatic disease showed that employment in a workplace where workplace support needs were met or exceeded was associated with a lower likelihood of reporting lost productivity when compared to those employed in a workplace where support needs were unmet [10]. While a supportive work environment has been associated with improved employee work outcomes over time in general population samples [21], very little longitudinal research has examined whether they can mitigate lost productivity among young adults with a rheumatic disease and concomitant mental health challenges.

One in five Canadians over the age of 15 have a rheumatic condition [22]. Population data show that adults living with rheumatic disease experience high rates of depression (~23%) when compared to those without rheumatic disease (~11%) [23]. Given the epidemiology of depressive disorders, the prevalence of depression among those living with a rheumatic condition may be particularly pronounced in young adulthood, especially among those not employed [24]. Individuals with more severe symptoms or disability due to their rheumatic disease may have higher levels of depression. Population-level studies also show that for young adults living with rheumatic disease, depressive symptoms are significantly associated with not participating in employment [4, 24–29]. However, little research has shown if, and how, workplace support can modify the association between depression morbidity and work productivity among those with rheumatic disease. Our study investigated the association of severe rheumatic disease symptoms and depressive symptom comorbidity on work productivity. We also examined whether workplace supports modified the relationship between severe rheumatic disease symptom severity and depressive symptoms on at-work productivity.

## Participants and Methods

Three waves of longitudinal data were analyzed from an ongoing online survey of a cohort of Canadian young adults with rheumatic disease (such as ankylosing spondylitis; juvenile ankylosing spondylitis; juvenile idiopathic arthritis; juvenile rheumatoid arthritis; systemic lupus erythematosus; osteoarthritis; psoriatic arthritis; rheumatoid arthritis; scleroderma; and/or seronegative enthesitis). Participants were recruited from three sources: rheumatology clinics in three Canadian provinces, a survey research firm, and community-based organizations [30]. Participants in the baseline survey were 18 to 35 years old, reported a physician-diagnosed rheumatic disease, and had paid employment at some point in the past year. All eligible participants were provided with detailed study information and a link to the survey. Informed consent was obtained from all participants. The baseline survey was conducted during the winter of 2019; each additional survey was conducted every subsequent 9 months (27-month total follow-up). The online surveys collected information on sociodemographic and rheumatic disease/health factors, employment experiences, and the role of workplace supports. Additional details regarding participant recruitment and survey development have been described elsewhere [10, 30]. Study procedures were approved by the University of Toronto Research Ethics Board (REB# 36588).

In the first wave, 133 participants were recruited. To be included in the analytic study sample, participants needed to have completed all three surveys (n = 118) and be employed at each survey wave. Of these participants, 64% (n = 76) reported being employed at each survey wave and composed the analytic sample for this study. Compared to those employed at all three survey cycles, participants who were not employed at all three surveys of the 27-month follow-up were more likely to be younger, have less than a post-secondary degree, be unmarried, have a personal income < $20,000, have childcare responsibilities, and work part-time at the initial baseline survey.

### Measures

#### Exposure: Morbidity

##### Severe Rheumatic Disease Symptoms

Participants were asked to rate the severity of pain, fatigue, and disease activity on an 11-point visual analog scale (0 = no pain/fatigue/disease activity; 10 = worst possible pain/fatigue/disease activity) [31]. Participants were defined as having severe rheumatic disease symptoms if they met one of the following three criteria: a pain score ≥ 6/10, a fatigue score ≥ 5/10, or a disease activity score ≥ 5/10. Cut-offs were established based on past studies conducted with this cohort [10].

##### Depressive Symptoms

Using the 2-item Patient Health Questionnaire (PHQ-2), participants were asked if over the past two weeks, they experienced little interest or pleasure in doing things or felt down, depressed, or hopeless. Response options were not at all, several days, more than half the days, or nearly every day. Scores ranged from 0 to 8, with a score of ≥ 3 indicating potential depressed mood. The PHQ-2 has shown to have high sensitivity and specificity in Canadian samples of those over age 18 years with rheumatic arthritis [32, 33].

To define morbidity level, a variable was created based on whether a participant reported no severe rheumatic disease symptoms or depressive symptoms (no symptom group), either severe rheumatic disease symptoms or depressive symptoms (single morbidity group), or both severe rheumatic disease symptoms and depressive symptoms (comorbid group).

#### Outcome Measures

##### Presenteeism

Using a global item from the Work Productivity and Activity Impairment Questionnaire, participants were asked, “During the past seven days, how much did your health affect your productivity while you were working?” Response options used an 11-point scale (ranging from 0 to 10); higher scores indicated greater presenteeism [34].

##### Absenteeism

Participants were asked “in the past 3 months, how many missed workdays have you had as a result of your rheumatic disease?” Based on responses, participants were categorized as no workdays missed or missed one or more workdays [2].

#### Modifier Variable: Workplace Support

A list of 13 job accommodations (e.g., workstation adaptations, job modifications, schedule flexibility) and benefits (e.g., prescription drug coverage) were presented to participants to assess the availability of workplace supports. Participants were asked whether they needed the different workplace support (yes/no) and if they had used them (yes/no). These variables were selected based on prior quantitative and qualitative research conducted by the investigative team of the experience using job accommodations, adaptations, and extended benefits shown to be associated with presenteeism and absenteeism among individuals with rheumatic conditions [18–20]. A two-level variable was constructed for each survey wave: (1) unmet workplace support needs (i.e., a participant’s need for workplace supports was greater than their use of available workplace supports); and (2) workplace support needs met or exceeded (i.e., a participant’s need for workplace support was equal to what they used or they used more of available workplace support than they reported needing) [10].

#### Covariates

Participant demographic factors were used as covariates (Supplemental Table 1). These included age (18 to 25 years; 26 to 36 years), sex/gender (man; woman or other gender), industry employed (healthcare and social services; other industries, e.g., financial, government, technology, professional services, education, arts/culture, not for profit, utilities, sales, construction, manufacturing, agriculture, mining, forestry), rheumatic disease onset (pediatric rheumatic disease onset at < 18 years; adult rheumatic disease onset $\ge$ 18), childcare responsibilities (yes; no), highest level of education (< post-secondary educational attainment; $\ge$ post-secondary educational attainment or university), job tenure (years), and full- or part-time employment. Covariates at each survey wave generally remained constant, so only baseline covariates were used in analyses.

### Statistical Analyses

Descriptive analyses were conducted to summarize participant characteristics, examine variable distributions, and facilitate model building. We estimated the prevalence of morbidity on presenteeism and absenteeism over the study period. Chi-Square and ANOVA tests were used to examine the association between the variables by morbidity level. Pearson and Spearman correlation coefficients were estimated to understand the extent to which the prevalence of severe rheumatic disease symptoms and/or depressive symptoms, presenteeism, absenteeism, and workplace support changed over the three survey cycles.

To address the study objectives, two generalized estimating equations (GEE) were used to estimate the association between morbidity level and presenteeism (model 1) and absenteeism (model 2). Each model included the following variables: time (defined as survey waves at baseline, 2, or 3), morbidity level, workplace support at baseline, and covariates. Each GEE also included four cross-product terms which included time*morbidity level, time*workplace support, morbidity level*workplace support, and time*morbidity*supportive work environment. In model 1, a normal distribution and unstructured correlation matrix were used to estimate presenteeism. In model 2, a binomial distribution and unstructured correlation matrix were used to estimate absenteeism. Sensitivity analysis was conducted to further understand if specific accommodations and benefits moderated the association between morbidity level and productivity. Three policy-related workplace supports with high need reported in the cohort—availability of prescription drug coverage, health benefit coverage, and paid sick leave—were tested as effect modifiers. All statistical tests were conducted with an α level of 0.05. Analyses were completed in RStudio (version 4.3.1) [35].

## Results

### Participant Description

Similar percentages of participants were in the no severe rheumatic disease or depressive symptoms group (no morbidity group, 22.4%) and the severe rheumatic disease and depressive symptom group (comorbidity group, 22.4%), while 55.2% of participants reporting either severe rheumatic disease symptoms or depressive symptoms (single morbidity group). Table 1 describes the distribution of presenteeism, absenteeism, workplace support, and covariates at baseline for all participants and stratified by comorbidity level. Across all three morbidity groups, a greater proportion of participants were ages 26 to 36 years old at the first survey (77.6%), had a household income $20,000–69,999 (57.8%), had rheumatic disease onset in childhood (68.4%), did not have childcare responsibilities (94.7%), were employed full-time (working 35 h or more per week, 73.7%), and were employed in the healthcare/social services industries (59.2%). Among the single morbidity group, participants reporting severe depressive symptoms and not severe rheumatic disease were more likely to be men, less likely to be married, more likely to work in the healthcare/social services sector and indicated a longer median job tenure when compared to those in the no morbidity group.

**Table 1:** Cohort characteristics, stratified by morbidity level

	Morbidity level				
	Overall Cohort (n = 76)	No symptoms (n = 17)	Single morbidity^†^ (n = 42)	Comorbid^††^ (n = 17)	p
	n (%)/median [IQR]	n (%)/median [IQR]	n (%)/median [IQR]	n (%)/median [IQR]	
Age					
18–25 years	17 (22.4)	4 (23.5)	9 (21.4)	4 (23.5)	0.98
26–36 years	59 (77.6)	13 (76.5)	33 (78.6)	13 (76.5)	
Gender					
Man	12 (15.8)	2 (11.8)	5 (11.9)	5 (29.4)	0.22
Woman or Other Gender	64 (84.2)	15 (88.2)	37 (88.1)	12 (70.6)	
Educational attainment					
Post-Secondary Degree	52 (68.4)	8 (47.1)	32 (76.2)	12 (70.6)	0.09
Less than Post-Secondary Degree/Other	24 (31.6)	9 (52.9)	10 (23.8)	5 (29.4)	
Marital status					
Married or living as married	32 (42.1)	7 (41.2)	21 (50.0)	4 (23.5)	0.18
Divorced, widowed, or never married	44 (57.9)	10 (58.8)	21 (50.0)	13 (76.5)	
Household income					
Less than $20,000	8 (10.5)	2 (11.8)	4 (9.5)	2 (11.8)	0.75
$20,000–$69,999	44 (57.9)	9 (52.9)	25 (59.5)	10 (58.8)	
$70,000 and over	20 (26.3)	6 (35.3)	11 (26.2)	3 (17.6)	
Missing	4 (5.3)	0 (0.0)	2 (4.8)	2 (11.8)	
Childcare responsibilities					
Yes	4 (5.3)	0 (0.0)	3 (7.1)	1 (5.9)	0.53
No	72 (94.7)	17 (100.0)	39 (92.9)	16 (94.1)	
Rheumatic disease onset age					
Adulthood onset (18 years)	24 (31.6)	6 (35.3)	13 (31.0)	5 (29.4)	0.93
Childhood onset (< 18 years)	52 (68.4)	11 (64.7)	29 (69.0)	12 (70.6)	
Industry employment					
Healthcare/Social services	45 (59.2)	9 (52.9)	24 (57.1)	12 (70.6)	0.53
Other professions	31 (40.8)	8 (47.1)	18 (42.9)	5 (29.4)	
Full- vs. part-time employment					
Full-time ($$\ge$$ 35 h/week)	56 (73.7)	14 (82.4)	29 (69.0)	13 (76.5)	0.55
Part-time (< 35 h/week)	20 (26.3)	3 (17.6)	13 (31.0)	4 (23.5)	
Hours worked during an average week					
< 10 h	5 (6.6)	2 (11.8)	2 (4.8)	1 (5.9)	0.312
10 to 19 h	3 (3.9)	0 (0.0)	3 (7.1)	0 (0.0)	
20 to 29 h	6 (7.9)	1 (5.9)	5 (11.9)	0 (0.0)	
30 to 34 h	6 (7.9)	0 (0.0)	3 (7.1)	3 (17.6)	
> 35 h	56 (73.7)	14 (82.4)	29 (69.0)	13 (76.5)	
Job tenure (years)	2.00 [1.00, 5.25]	2.00 [1.00, 4.00]	2.00 [1.00, 4.75]	5.00 [1.00, 8.00]	0.20
Workplace supports available	7.00 [5.00, 10.00]	7.00 [6.00, 11.00]	7.00 [5.00, 10.00]	8.00 [5.00, 10.00]	0.95
Workplace support needs met					0.03
Workplace support needs met or exceeded	28 (36.8)	11 (64.7)	12 (28.6)	5 (29.4)	
Workplace support needs not met	48 (63.2)	6 (35.3)	30 (71.4)	12 (70.6)	
Presenteeism (median, [IQR])	4.00 [3.00, 7.00]	2.00 [1.00, 4.00]	5.50 [3.00, 7.00]	5.00 [3.00, 9.00]	< 0.01
Absenteeism					
No	34 (44.7)	12 (70.6)	18 (42.9)	4 (23.5)	0.02
Yes	42 (55.3)	5 (29.4)	24 (57.1)	13 (76.5)	

*IQR* interquartile range

^†^Single morbidity group includes those reporting either severe rheumatic disease symptoms or depressive symptoms

^††^Comorbid group includes those reporting both severe rheumatic disease symptoms and depressive symptoms

### Workplace Support

One-third of all participants (36.8%) reported that their workplace support needs were met or exceeded. Those in the no morbidity group were more likely to report that their needs were meet or exceeded (64.7%) compared to those in the single severe disease group (28.6%) or the group with both severe rheumatic disease and severe depressive symptoms (29.4%). Table 2 highlights the specific workplace support availability and need at baseline, by comorbidity level. For most participants across morbidity levels, nearly all workplace supports were available at their workplaces. Most participants in all three morbidity groups reported often or always needing prescription drug coverage, healthcare benefit, and other workplace support benefits. Participants in the comorbid group reported always or often needing work schedule flexibility (52.9%), which was higher than the one morbidity (31.0%) and no morbidity groups (17.6%).

**Table 2:** Workplace support and availability at baseline, stratified by morbidity level

	Workplace Support Availability				Workplace Support Need		
	No symptoms	Single morbidity	Co morbid		No symptoms	Single morbidity	Co morbid
	(n = 17)	(n = 42)	(n = 17)		(n = 17)	(n = 42)	(n = 17)
Prescription Drug Coverage				Prescription Drug Coverage			
Available	14 (82.4)	32 (76.2)	13 (76.5)	Never/Infrequently Needed	3 (17.6)	5 (11.9)	2 (11.8)
Not Available	3 (17.6)	7 (16.7)	4 (23.5)	Sometimes Needed	2 (11.8)	9 (21.4)	1 (5.9)
Unknown	0 (0.0)	3 (7.1)	0 (0.0)	Often/Always Needed	12 (70.6)	28 (66.7)	14 (82.4)
Healthcare Benefits				Healthcare Benefits			
Available	14 (82.4)	31 (73.8)	11 (64.7)	Never/Infrequently Needed	5 (29.4)	8 (19.0)	3 (17.6)
Not Available	3 (17.6)	8 (19.0)	6 (35.3)	Sometimes Needed	2 (11.8)	9 (21.4)	4 (23.5)
Unknown	0 (0.0)	3 (7.1)	0 (0.0)	Often/Always Needed	10 (58.8)	25 (59.5)	10 (58.8)
Paid Sick Leave				Paid Sick Leave			
Available	12 (70.6)	26 (61.9)	14 (82.4)	Never/Infrequently Needed	13 (76.5)	17 (40.5)	6 (35.3)
Not Available	3 (17.6)	11 (26.2)	3 (17.6)	Sometimes Needed	1 (5.9)	8 (19.0)	4 (23.5)
Unknown	2 (11.8)	5 (11.9)	0 (0.0)	Often/Always Needed	3 (17.6)	17 (40.5)	7 (41.2)
Employee Assistance Program				Employee Assistance Program			
Available	11 (64.7)	25 (59.5)	10 (58.8)	Never/Infrequently Needed	15 (88.2)	34 (81.0)	8 (47.1)
Not Available	4 (23.5)	9 (21.4)	6 (35.3)	Sometimes Needed	1 (5.9)	4 (9.5)	4 (23.5)
Unknown	2 (11.8)	8 (19.0)	1 (5.9)	Often/Always Needed	1 (5.9)	4 (9.5)	5 (29.4)
Work Schedule Flexibility				Work Schedule Flexibility			
Available	14 (82.4)	30 (71.4)	13 (76.5)	Never/Infrequently Needed	10 (58.8)	7 (16.7)	7 (41.2)
Not Available	2 (11.8)	9 (21.4)	3 (17.6)	Sometimes Needed	4 (23.5)	22 (52.4)	1 (5.9)
Unknown	1 (5.9)	3 (7.1)	1 (5.9)	Often/Always Needed	3 (17.6)	13 (31.0)	9 (52.9)
Modified Job Duties				Modified Job Duties			
Available	7 (41.2)	25 (59.5)	10 (58.8)	Never/Infrequently Needed	15 (88.2)	24 (57.1)	14 (82.4)
Not Available	3 (17.6)	6 (14.3)	6 (35.3)	Sometimes Needed	2 (11.8)	12 (28.6)	1 (5.9)
Unknown	7 (41.2)	11 (26.2)	1 (5.9)	Often/Always Needed	0 (0.0)	6 (14.3)	2 (11.8)
Work From Home Arrangements				Work From Home Arrangements			
Available	7 (41.2)	18 (42.9)	7 (41.2)	Never/Infrequently Needed	12 (70.6)	19 (45.2)	12 (70.6)
Not Available	7 (41.2)	19 (45.2)	8 (47.1)	Sometimes Needed	2 (11.8)	16 (38.1)	1 (5.9)
Unknown	3 (17.6)	5 (11.9)	2 (11.8)	Often/Always Needed	3 (17.6)	6 (14.3)	4 (23.5)
Accessible Workplace				Accessible Workplace			
Available	10 (58.8)	24 (57.1)	10 (58.8)	Never/Infrequently Needed	16 (94.1)	29 (69.0)	12 (70.6)
Not Available	2 (11.8)	11 (26.2)	5 (29.4)	Sometimes Needed	0 (0.0)	5 (11.9)	2 (11.8)
Unknown	5 (29.4)	7 (16.7)	2 (11.8)	Often/Always Needed	1 (5.9)	8 (19.0)	3 (17.6)
Workstation Modifications				Workstation Modifications			
Available	8 (47.1)	26 (61.9)	10 (58.8)	Never/Infrequently Needed	12 (70.6)	22 (52.4)	11 (64.7)
Not Available	8 (47.1)	10 (23.8)	7 (41.2)	Sometimes Needed	3 (17.6)	9 (21.4)	1 (5.9)
Unknown	1 (5.9)	6 (14.3)	0 (0.0)	Often/Always Needed	2 (11.8)	11 (26.2)	5 (29.4)
Assistive Devices/Technology				Assistive Devices/Technology			
Available	8 (47.1)	15 (35.7)	8 (47.1)	Never/Infrequently Needed	15 (88.2)	33 (78.6)	12 (70.6)
Not Available	5 (29.4)	11 (26.2)	9 (52.9)	Sometimes Needed	1 (5.9)	4 (9.5)	2 (11.8)
Unknown	4 (23.5)	16 (38.1)	0 (0.0)	Often/Always Needed	1 (5.9)	5 (11.9)	3 (17.6)
At-work Facilities to Manage Health				At-work Facilities to Manage Health			
Available	9 (52.9)	20 (47.6)	11 (64.7)	Never/Infrequently Needed	14 (82.4)	29 (69.0)	12 (70.6)
Not Available	4 (23.5)	11 (26.2)	4 (23.5)	Sometimes Needed	2 (11.8)	7 (16.7)	3 (17.6)
Unknown	4 (23.5)	11 (26.2)	2 (11.8)	Often/Always Needed	1 (5.9)	6 (14.3)	2 (11.8)
Informal Modifications of Work				Informal Modifications of Work			
Available	9 (52.9)	24 (57.1)	8 (47.1)	Never/Infrequently Needed	15 (88.2)	24 (57.1)	12 (70.6)
Not Available	4 (23.5)	6 (14.3)	6 (35.3)	Sometimes Needed	1 (5.9)	9 (21.4)	2 (11.8)
Unknown	4 (23.5)	12 (28.6)	3 (17.6)	Often/Always Needed	1 (5.9)	9 (21.4)	3 (17.6)
Other Workplace Supports				Other Workplace Supports			
Available	1 (5.9)	2 (4.8)	1 (5.9)	Never/Infrequently Needed	15 (88.2)	32 (76.2)	13 (76.5)
Not Available	5 (29.4)	13 (31.0)	10 (58.8)	Sometimes Needed	0 (0.0)	6 (14.3)	0 (0.0)
Unknown	11 (64.7)	27 (64.3)	6 (35.3)	Often/Always Needed	2 (11.8)	4 (9.5)	4 (23.5)

### Productivity

The median presenteeism score at baseline was 4 out of a possible ten points (interquartile range [IQR] = 3–7, Table 1). Participants in the no morbidity group reported the lowest presenteeism (median = 2; IQR = 1–4) when compared to participants in the single morbidity group (median = 6; IQR = 3–7;) and for participants reporting comorbidity (median = 5; IQR = 3–9) (Table 1). Over half of participants reported absenteeism at baseline (55.3%). The percentage of participants in the no morbidity group reporting absenteeism was lower (29.4%) compared to those in the single morbidity group absenteeism (57.1%) or the comorbidity group (76.5%).

Table 3 highlights the relationship between morbidity level, presenteeism, absenteeism, and workplace support over time. A small-to-moderate positive correlation existed among morbidity level across time, suggesting that most participants reported similar morbidity across the three survey waves. Similarly, a positive correlation was seen between morbidity and presenteeism over time. A somewhat weaker positive correlation was seen between morbidity and absenteeism over time. There were small negative correlations seen between workplace support and morbidity over time, suggesting that as morbidity increased, workplace support needs were unmet.

**Table 3:** Correlations between exposure, moderator, and outcome over time

	Morbidity Level, T1	Morbidity Level, T2	Morbidity Level, T3	Presenteeism, T1	Presenteeism, T2	Presenteeism, T3	Absenteeism, T1	Absenteeism, T2	Absenteeism, T3	Workplace supports, T1	Workplace supports, T2	Workplace supports, T3
Morbidity level, T1	1.00											
Morbidity level, T2	**0.62**	1.00										
Morbidity level, T3	**0.47**	**0.41**	1.00									
Presenteeism, T1	**0.41**	**0.46**	**0.33**	1.00								
Presenteeism, T2	**0.47**	**0.60**	**0.37**	**0.65**	1.00							
Presenteeism, T3	**0.33**	**0.40**	**0.55**	**0.46**	**0.53**	1.00						
Absenteeism, T1	0.30	0.31	0.48	0.16	0.06	0.07	1.00					
Absenteeism, T2	0.18	**0.35**	0.27	**0.53**	**0.55**	**0.24**	0.08	1.00				
Absenteeism, T3	0.21	0.31	0.47	0.18	0.20	**0.42**	0.08	0.06	1.00			
Workplace supports, T1	**− 0.24**	**− 0.26**	**− 0.29**	**− 0.37**	**− 0.39**	**− 0.26**	**− **0.29	**− 0.20**	**− **0.29	1.00		
Workplace supports, T2	**− 0.28**	**− 0.30**	**− 0.24**	**− 0.37**	**− 0.35**	**− 0.24**	**− **0.21	**− 0.21**	**− **0.13	**0.48**	1.00	
Workplace supports, T3	**− **0.13	**− **0.15	**− **0.16	**− **0.13	**− **0.20	**− **0.07	**− **0.21	**− **0.17	**− **0.12	**0.57**	**0.27**	1.00

Bolded values represent correlation coefficients with p values significant at the α = 0.05 level

**Fig. 1:**
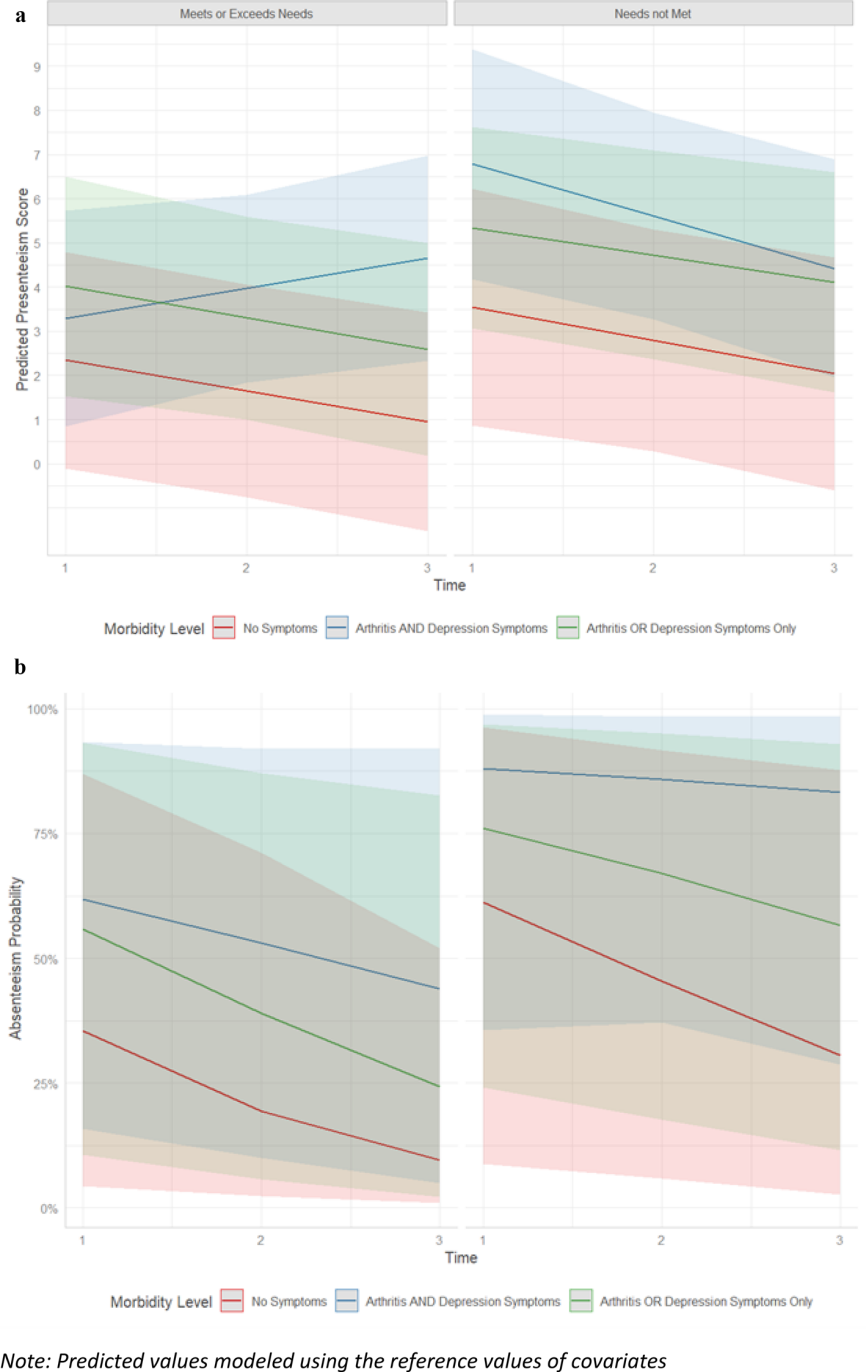
**A** Predicted values of presenteeism, by morbidity level and work accommodation support. **B** Predicted absenteeism probability, by morbidity level and work accommodation support. *Note* Predicted values modeled using the reference values of covariates

Table 4 and Figs. 1A and B summarize findings from the GEE models, where presenteeism (model 1) and absenteeism (model 2) were regressed on morbidity, workplace supports, the different covariates, and time. Among participants who reported that their workplace support needs were met at baseline, those with no morbidity or a single morbidity were more likely to experience decreased presenteeism over time (model 1). In contrast, among participants who reported workplace support needs were met or exceeded, those in the comorbid group indicated increasing presenteeism over time.

**Table 4:** Association between morbidity level and workplace support on presenteeism and absenteeism

	Presenteeism			Absenteeism		
Variable	β	95% CI^a^	p	OR^*1*^	95% CI^a^	p
Age						
18–25 years	–	–		–	–	
26–36 years	**− **0.09	**− **1.04, 0.87	> 0.90	0.97	0.38, 2.48	> 0.90
Gender						
Man	–	–		–	–	
Woman or Other Gender	**− **0.85	**− **2.15, 0.45	0.20	**0.18**	**0.05, 0.62**	**0.01**
Industry employed						
Healthcare/Social services	–	–		–	–	
Other professions	0.35	**− **0.52, 1.22	0.40	0.59	0.24, 1.40	0.20
Rheumatic disease onset age						
Adulthood onset (18 years)	–	–		–	–	
Childhood onset (< 18 years)	0.06	**− **0.72, 0.84	0.90	1.52	0.68, 3.41	0.30
Childcare responsibilities						
Yes	–	–		–	–	
No	0.28	**− **1.56, 2.11	0.80	2.22	0.53, 9.35	0.30
Educational attainment						
Post-Secondary Degree	–	–		–	–	
Less than Post-Secondary Degree/Other	0.17	**− **0.69, 1.03	0.70	1.49	0.61, 3.60	0.40
Job Tenure (years)	0.04	**− **0.08, 0.16	0.50	0.90	0.80, 1.02	0.09
Full- vs. Part-Time Employment						
Full-time ($\ge$ 30 h/week)	–	––		–	–	
Part-time (< 30 h/week)	**1.40**	**0.45, 2.26**	**0.01**	1.15	0.49, 2.74	0.70
Accommodations Available at Work	0.09	**− **0.02, 0.20	0.11	**1.13**	**1.01, 1.26**	**0.04**
Time	**− 0.69**	**− 1.23, − 0.16**	**0.01**	**0.44**	**0.23, 0.83**	**0.01**
Morbidity Level						
No Symptoms	–	–		–	–	
Single morbidity	**− **0.44	**− **3.23, 2.34	0.80	1.86	0.13, 26.7	0.60
Comorbidity	1.70	**− **0.57, 3.97	0.14	2.01	0.17, 23.8	0.60
Workplace Support						
Workplace support needs met or exceeded	–	–		–	–	
Workplace support needs not met	1.30	**− **1.22, 3.76	0.30	2.37	0.12, 48.2	0.60
Time * Morbidity Level						
Time * Comorbidity	**1.40**	**0.21, 2.55**	**0.02**	1.59	0.56, 4.55	0.40
Time * Single morbidity	**− **0.02	**− **1.05, 1.00	> 0.90	1.15	0.37, 3.58	0.80
Time * Supportive Work Environment						
Time * Workplace support needs not met	**− **0.06	**− **1.1, 0.94	> 0.90	1.21	0.36, 4.02	0.80
Morbidity Level * Supportive Work Environment						
Comorbidity * Workplace support needs not met	**4.10**	**0.02, 8.19**	**0.05**	1.60	0.02, 114	0.80
Single morbidity * Workplace support needs not met	**− **0.04	**− **3.18, 3.10	> 0.90	0.83	0.02, 32.3	> 0.9
Time * Morbidity Level * Workplace supports						
Time * Comorbidity * Workplace support needs not met	**− 1.80**	**− 3.50, − 0.13**	**0.04**	0.98	0.18, 5.38	> 0.90
Time * Single morbidity * Workplace support needs not met	0.16	**− **1.22, 1.54	0.80	1.05	0.20, 5.45	> 0.90

^a^*OR* odds ratio, *CI* confidence interval

– Reference Level Bolded values significant at the α = 0.05 level

Findings from model 2 (absenteeism) indicated that the interaction between time, morbidity, and workplace was not statistically significant. Accordingly, we describe the main effects of time, morbidity, and workplace support. Among participants reporting that workplace support needs were met or exceeded, the probability of reporting absenteeism decreased for participants across all morbidity groups over time. Participants who reported comorbidity had a greater probability of absenteeism when compared to participants no morbidity or a single morbidity. Participants who reported that their workplace supports were met or exceeded had lower odds of absenteeism when compared to those with unmet workplace support needs. For our sensitivity analysis, when exploring the specific role of prescription drug coverage, healthcare benefit, and paid sick leave as modifiers in the sensitivity analysis, similar findings were seen in that participants reported lower odds of absenteeism.

## Discussion

Using a longitudinal design, our study is one of the first to demonstrate that comorbid severe rheumatic disease symptoms and depressive symptoms among young adults can have a pronounced negative association on work productivity over time. Findings also highlight the role of a supportive workplace and addressing lost productivity including absenteeism and presenteeism. These findings highlight the importance of designing and implementing strategies within the work environment that prevents work disability for young people living with rheumatic disease and continuing to identify approaches to address presenteeism among those living with both rheumatic disease and depressive symptoms.

Over one-fifth of young adults in our study reported comorbid rheumatic disease symptoms and depressive symptoms. Research has indicated that having a rheumatic disease and depressive symptoms is associated with a higher likelihood of not working [36]. Given the potential work-related consequences of comorbidity, being able to discriminate different levels of symptom severity is vital. In our study, we define comorbidity as if a participant had noticeable rheumatic symptoms (characterized by severe fatigue, pain, or disease activity), and/or depressive symptoms (as defined by the PHQ-2). While our definition is based on selected measures, at each survey rheumatic disease symptoms (*r* ~0.50–0.75, data available from authors) were moderately correlated, suggesting that for most participants, high scores on one measure were correlated with high scores on the other two measures. Our definition was able to discriminate across different employment experiences and outcomes. To expand on our research, detailed questions could be used to classify comorbidity with greater specificity.

Those with comorbidity were more likely to report greater presenteeism and a higher likelihood of absenteeism when compared to the no morbidity group, which aligns with past research [37]. Notably, our findings indicated that both presenteeism and likelihood of reporting absenteeism increased for young adults with comorbid severe rheumatic disease and depressive symptoms. Growing research indicates that many employers have limited experience in designing and implementing supports for workers living with rheumatic disease, depression, and other chronic health conditions [38, 39]. Our study describes productivity challenges among a particularly vulnerable group of young workers that have the potential to have an acute impact employment at the early career phase, and lasting economic impact for the remainder of their working lives. Given the pronounced burden of depression among young adults with rheumatic arthritis [23], our study underscores the importance of strategies to reduce the impact of mental health symptoms—such as increased awareness about depression and psychological hazards in the workplace, access to flexible work arrangements, and availability of employee assistance programs—should be coupled with efforts to reduce arthritis-related work disability [4, 38].

Our research adds to a growing literature on the role of the work environment, particularly the provision of supports, as a critical contextual factor that can impact the work productivity of people living with rheumatic disease. Young adults living with rheumatic disease report needing workplace supports to provide material and psychosocial resources, accommodations, and workplace policies to manage health challenges, and navigate job demands and challenges at the early career phase [18, 20]. In our study, compared to participants with no morbidity, a higher proportion of participants with morbidity from depression and severe rheumatic arthritis symptoms had a high need for paid sick leave, employee assistance programs, work schedule flexibility, workstation modifications, work from home arrangements, and informal modifications to work. These workplace supports could be beneficial to young workers to manage the demands of comorbid rheumatic disease and depressive symptoms. Employers can play in important role in promoting sustained work productivity among young adults living with rheumatic disease.

Interestingly, for participants with comorbid severe rheumatic disease and depressive symptoms who indicated that their workplace support needs were met or exceeded there was an increase in presenteeism. This seemingly paradoxical finding may be because, despite having support needs met (or even exceeded) in their job, these individuals were still having to manage both depressive and severe rheumatic symptoms at work. This “double whammy” of rheumatic disease and depression symptoms may have interfered with a potentially wider range of job demands that went beyond the physical demands of work, such as cognitive and interpersonal demands [40]. As a result, the likelihood of presenteeism may have been greater. Despite this, we found that these participants were able to continue working, which may be due, in part, to the workplace supports they received despite comorbidity [41]. Conversely, growing evidence suggests that there may be positive effects of presenteeism (such as building self-efficacy from being able to work, despite having a chronic condition), making workers view presenteeism in a more positive light [42]. Offering workplace supports tailored to the needs of young adults with rheumatic disease when entering their careers may help these workers sustain employment, especially if workplace support is coupled with pharmacological and non-pharmacological interventions and treatment.

Our study is novel in that we used an analytic sample from a Canadian cohort of employed young adults with rheumatic disease over a 27-month period who were recruited from the community and rheumatology clinics. We build on past cross-sectional research which has indicated an association between more severe rheumatic disease and mental health symptoms and lost productivity and work disability in young adulthood. Through our longitudinal design, our findings provide temporal evidence of a relationship severe rheumatic disease symptoms and greater absenteeism and presenteeism. Also, our research enabled us to better understand the role workplace supports can have on productivity.

Our findings should be interpreted with the following limitations in mind. The analytical sample size of the cohort was relatively small, which may have led to reduced statistical power to uncover associations in the interaction analysis, particularly for absenteeism. Recruiting large samples of young adults with rheumatoid arthritis in survey-based research is challenging. Studies of a similar nature have sample sizes ranging from 4 to 47 participants [43, 44].

The study used the two-item PHQ-2 measure as a proxy of depressive symptoms with high sensitivity and specificity of the PHQ-2 in studies of persons living with rheumatic disease [32]. Measuring depression through clinical diagnosis could have enabled to produce more accurate estimates of participants experiencing depressive symptoms. Survey waves were separated by 9 month. Accordingly, we were unable to measure the fluctuations in rheumatic disease symptom severity and depressive symptoms that may occur on a daily or weekly basis. If fluctuations occurred between survey waves, it is possible that findings may have underestimated the association between morbidity and lost productivity.

Comorbid severe rheumatic disease symptoms and depressive symptoms can have an impact on presenteeism and absenteeism among young adults. Providing a supportive work environment represents one important strategy that can be taken to address lost productivity and to sustain employment, especially young adults reporting comorbidities. Findings have important implications for both clinical care teams and workplace stakeholders and bring greater awareness to how rheumatic disease and depression comorbidity may impact work productivity; how despite having illness, the role of work is a critical aspect of life for these young adults; and the need for this population to have a work environment that is supportive of their complex health needs associated with their health conditions. Creating a supportive work environment can have long-lasting benefits for young adults with rheumatic disease as they transition into the world of work and move into different phases of their career and may manage changes to their mental health.

## Supplementary Information

Below is the link to the electronic supplementary material.

Supplementary file1 (DOCX 18 KB)

## Data Availability

No datasets were generated or analysed during the current study.
