# Supplementary material for: Double Trouble! Do Workplace Supports Mitigate Lost Productivity for Young Workers with Both Severe Rheumatic Diseases and Depressive Symptoms?
Source: J Occup Rehabil. 2024 Jul 3;35(3):491–504. doi: 10.1007/s10926-024-10217-8 (PMC12361260; doi:10.1007/s10926-024-10217-8)
Supplement: Supplementary file 1 — Supplementary file1 (DOCX 18 KB) [file 10926_2024_10217_MOESM1_ESM.docx]

**Double trouble! Do workplace supports mitigate lost productivity for young workers with both severe rheumatic diseases and depressive symptoms?**

**Supplementary Information**

**May 23^rd^, 2024**

**Supplementary Table 1. Information on Key Study Measures**

| Variable | Role | Type | Categories or Range | Survey Measurements Used |
| --- | --- | --- | --- | --- |
| Rheumatic & depressive symptom morbidity | Exposure | Categorical | 0-No Symptom Group 1-Single Morbidity Group (Either rheumatic or depressive symptoms) 2-Comorbid Group (Both rheumatic and depressive symptoms) | T1, T2, T3 |
| Presenteeism | Outcome | Continuous | 0 – 11 | T1, T2, T3 |
| Absenteeism | Outcome | Binary | 0-No Workdays Missed; 1-Missed One of More Workdays | T1, T2, T3 |
| Workplace Support | Effect Modifier | Binary | 0-Workplace Support Needs met or exceeded; 1-Unmet Workplace Support Needs | T1, T2, T3 (Correlations) T1 (GEE models) |
| Age | Covariate | Binary | 0-18 to 25 years old; 1-26 to 36 years old | T1 |
| Sex/Gender | Covariate | Binary | 0-Man 1-Woman or Other Gender | T1 |
| Industry | Covariate | Categorical | financial, government, technology, professional services, education, arts/culture, not for profit, utilities, sales, construction, manufacturing, agriculture, mining, forestry | T1 |
| Rheumatic Disease Onset | Covariate | Binary | 0-Pediatric Rheumatic Disease Onset (<18 years old), 1 – Adult Rheumatic Disease Onset (>18 years old) | T1 |
| Childcare Responsibilities | Covariate | Binary | 0-No 1-Yes | T1 |
| Highest Level of Education | Covariate | Binary | 0- <post-secondary educational attainment; 1- ≥post-secondary educational attainment or university | T1 |
| Job tenure, years | Covariate | Continuous | 0 – 13 years | T1 |
| Full vs. Part Time Employment | Covariate | Binary | 0-Full time employment (> 35 hours), 1-Part time employment (<35 Hours) | T1 |
| Hours worked in an average week | Covariate (descriptive table only) | Categorical | 0- < 10 Hours  1- 10 to 19 Hours  2- 20 to 29 Hours  3- 30 to 34 Hours  4- > 35 Hours | T1 |
